# Purpura Fulminans Due to Neisseria meningitidis Septicemia Treated With Adjunctive Hyperbaric Oxygen Therapy: A Case Report

**DOI:** 10.7759/cureus.98817

**Published:** 2025-12-09

**Authors:** Noora Bani Hammad, Abdulla Alghatam, Jawaher Aljalahma, Noora Albuainain

**Affiliations:** 1 Medicine and Surgery, Bahrain Defence Force Hospital, Riffa, BHR; 2 Dermatology, Bahrain Defence Force Hospital, Riffa, BHR; 3 Dermatology, Salmaniya Medical Complex, Manama, BHR; 4 Surgery, Bahrain Defence Force Hospital, Riffa, BHR

**Keywords:** fulminans purpura, hyperbaric oxygen therapy (hbot), neisseria meningitidis (n. meningitidis), tissue necrosis, ­wound healing

## Abstract

*Neisseria meningitidis*, a pathogenic bacterium known for causing bacterial meningitis, can also lead to severe systemic infections, including septicaemia. Among the most severe manifestations of *N. meningitidis* septicaemia is purpura fulminans (PF), a life-threatening condition characterized by rapidly progressive skin necrosis and disseminated intravascular coagulation. We report a case of a previously healthy 25-year-old male who presented with an acute onset of fever, rigors, and a diffuse petechial rash. Within hours, the patient developed extensive PF, accompanied by signs of septic shock. Despite prompt initiation of broad-spectrum antibiotics and supportive care, the patient's condition rapidly deteriorated. Diagnostic evaluation revealed *N. meningitidis* as the causative agent of the septicaemia. The patient was treated with targeted antibiotic therapy and aggressive supportive measures. Despite these interventions, the patient experienced severe complications, including PF that ultimately required debridement and multiple hyperbaric oxygen therapy sessions, which preserved the patient's limbs. This case highlights the aggressive nature of *N. meningitidis* septicaemia associated with PF, emphasizing the need for early recognition and immediate intervention. It underscores the importance of rapid diagnostic and therapeutic strategies to manage this critical condition effectively. The case also illustrates the potential for rapid deterioration and the challenges in managing such severe presentations of meningococcal infection.

## Introduction

*Neisseria meningitidis* is a Gram-negative encapsulated diplococcus and a major cause of bacterial meningitis, which carries high morbidity and mortality. While meningococcal infections primarily present as meningitis, they can also manifest as severe systemic infections, including meningococcal septicaemia. Purpura fulminans (PF) is a rapidly progressing and potentially fatal condition characterized by widespread intravascular thrombosis, disseminated intravascular coagulation (DIC), and extensive skin necrosis. The condition often leads to multi-organ failure and requires immediate medical intervention [1]. Furthermore, PF is a rare manifestation amongst adults, with the majority of cases being cited as case reports and case series. It is seen in 10-20% of patients with meningococcal septicaemia, with the most common bacterial organisms being meningococcus and *Streptococcus pneumoniae*. In terms of the mortality rate, it is very high, going up to 60% in affected individuals [2].

The pathophysiology of PF involves the systemic activation of the coagulation cascade and subsequent vascular damage, resulting in a petechial rash that rapidly evolves into full-thickness skin necrosis and deep muscle damage [3]. Although relatively rare, the condition poses a significant challenge due to its acute presentation and the potential for rapid deterioration. *N. meningitidis* septicaemia associated with PF represents a medical emergency requiring swift action to improve patient outcomes. Treatment of PF focuses on promptly identifying and treating the underlying cause. This entails aggressive antibiotic therapy, fluid resuscitation, and supportive care in an intensive care setting. Protein C replacement, intravenous immunoglobulin (IVIG), anticoagulation, and hyperbaric oxygen therapy (HBOT) are all treatment options that can be considered in the management of PF. In severe cases, surgical interventions, including debridement, skin grafting, and amputation, may be resorted to in order to manage tissue damage [1,3]. This case report contributes to the understanding of this rare but severe manifestation, providing insights into the clinical management and potential outcomes of such a critical condition.

## Case presentation

**Figure 1:**
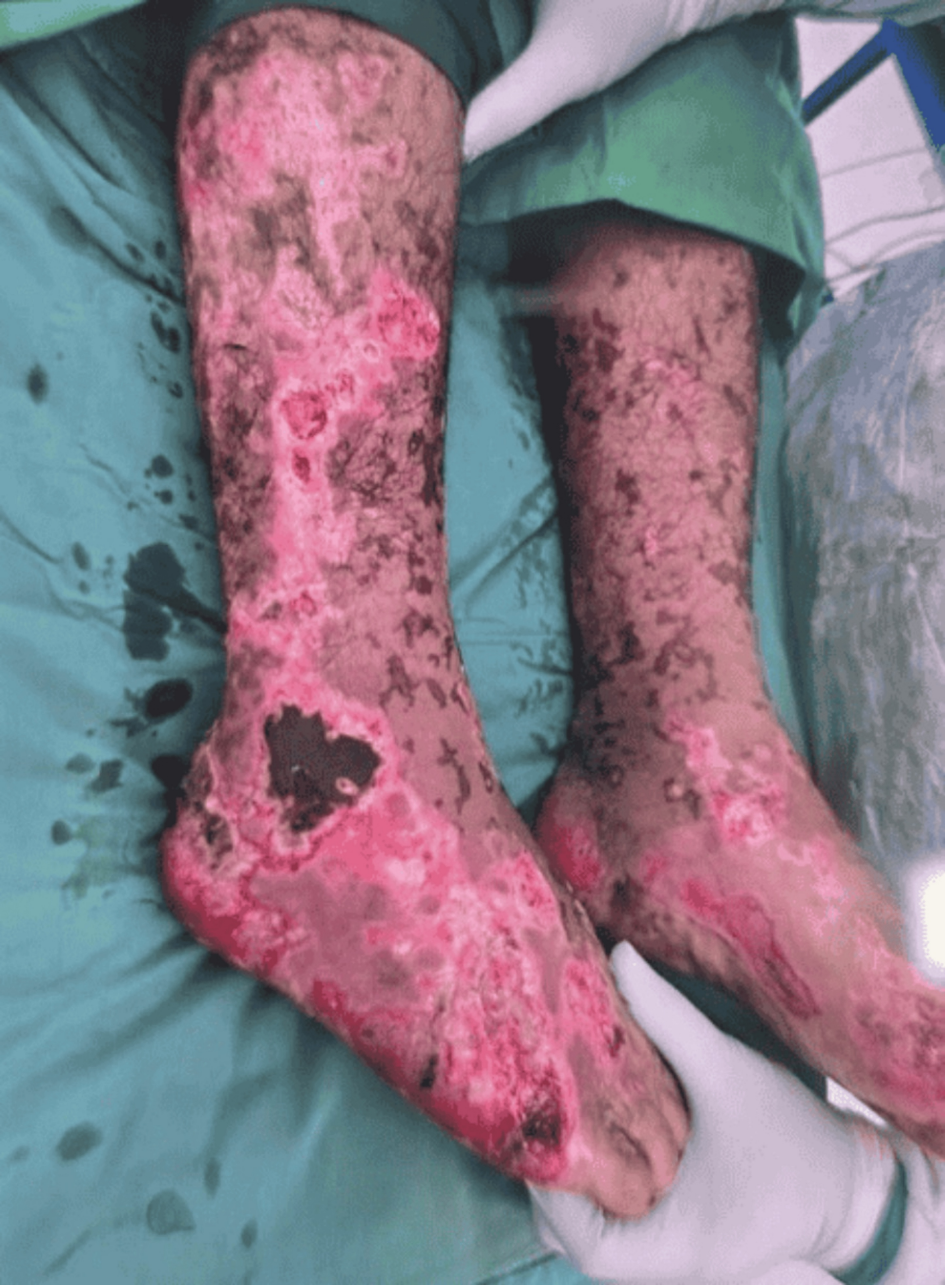
Extensive purpuric and hemorrhagic skin lesions involving the lower extremities at initial presentation, consistent with purpura fulminans secondary to septicemia.

**Figure 2:**
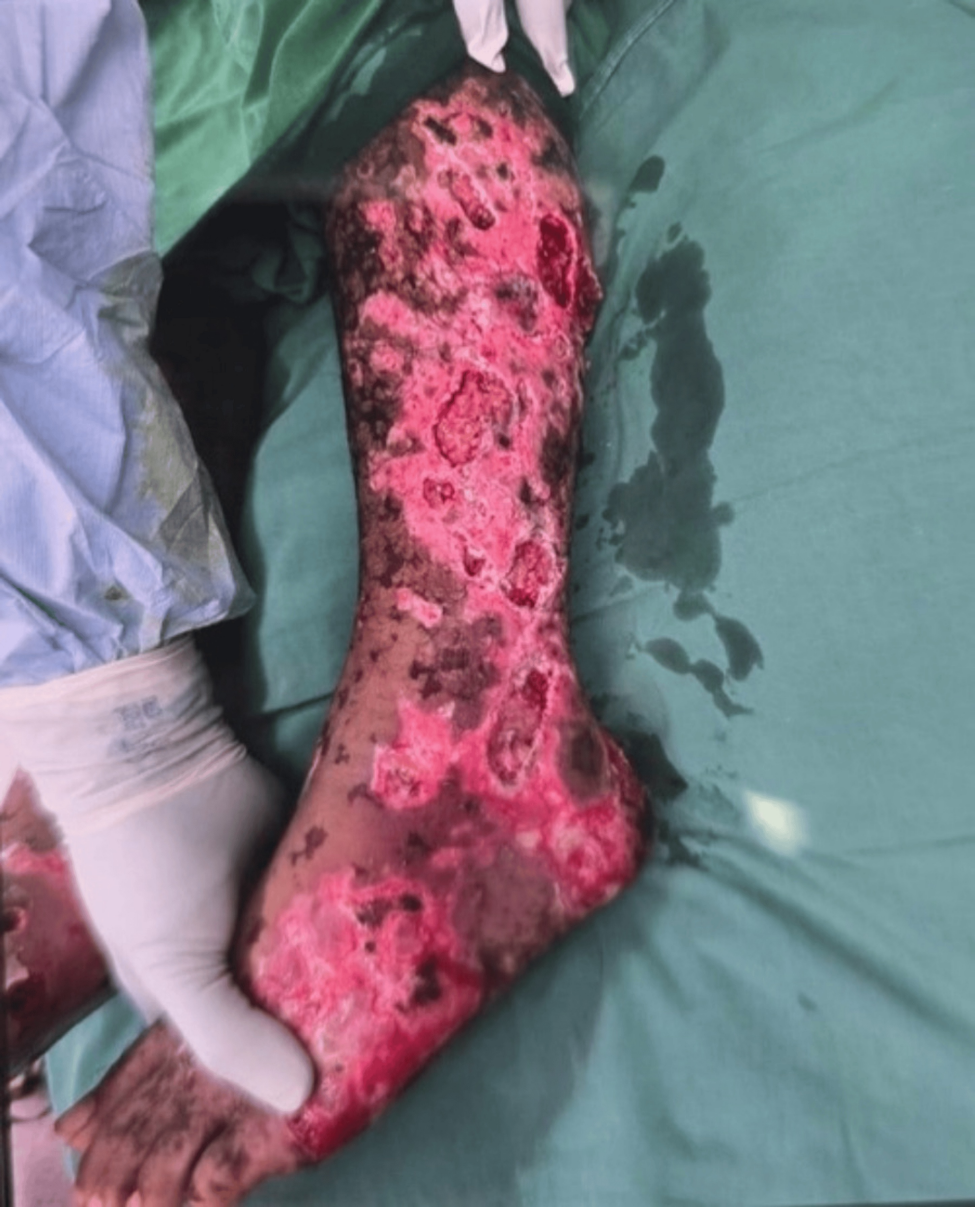
Extensive necrotic and ulcerative skin involvement of the lower limb and foot, demonstrating severe cutaneous tissue damage associated with purpura fulminans secondary to septicemia, prior to initiation of hyperbaric oxygen therapy.

**Figure 3:**
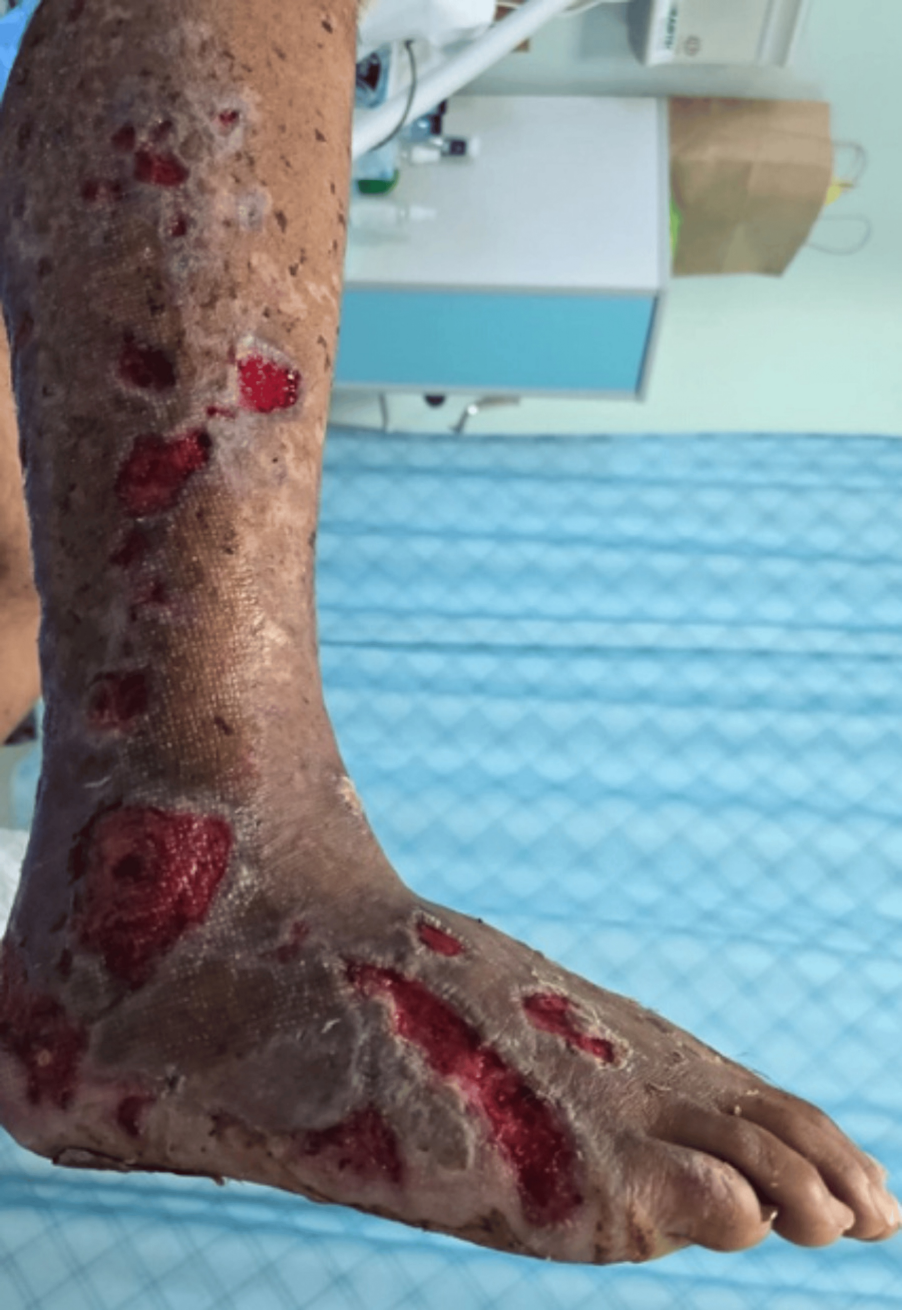
Clinical improvement of skin lesions following treatment, demonstrating partial resolution of purpura and stabilization of necrotic areas after adjunctive hyperbaric oxygen therapy.

A previously healthy 25-year-old male presented to the emergency department via air ambulance from Thailand with a fever and a necrotic rash that involved all limbs. He was last known to be well two weeks prior, when he was on vacation in Thailand. He developed a fever along with lesions that were initially maculopapular and concentrated over both the upper and lower limbs. Three days later, his fever and the lesions progressed to become ischemic and necrotic (Figures 1-3). He was admitted to the hospital overseas, where haemoculture showed *N. meningitidis*, and cerebrospinal fluid (CSF) latex agglutination was positive for *N. meningitidis* serogroup YW135.

On initial evaluation, his vitals were notable for a temperature of 39.4°C, heart rate of 109, respiratory rate of 22, and blood pressure of 90/68. His physical examination entailed a grossly normal cardiovascular, respiratory, and abdominal examination. Furthermore, his cranial nerve examination showed normal higher mental functions, and the sensory and motor examination was normal initially. Physical examination was also negative for the Brudzinski and Kernig signs and nuchal rigidity.

In terms of imaging, the chest X-ray was unremarkable, and ultrasound doppler for both lower limbs showed no evidence of deep vein thrombosis (DVT); however, there was mild diffuse subcutaneous edema across both lower limbs. Ultrasound abdomen showed splenomegaly (12.8 cm). Computed tomography angiography (CTA) of the abdomen was unremarkable, and CTA of the peripherals showed several intramuscular rim-enhancing lesions in both feet, diffuse dirty fat stranding of subcutaneous tissue of both lower legs, and markedly swollen subcutaneous tissue at the dorsal aspect of both feet, with no evidence of arterial occlusion or stenosis. As for the labs, they were notable for a platelet count of (102,000/Ul). He demonstrated low levels of protein C, protein S, and antithrombin III. Consequently, he received anticoagulants and antiplatelets; however, he did not receive protein C. During the patient's hospitalization abroad, he was started on broad-spectrum antibiotics, including tigecycline and ceftriaxone. He was on morphine and fentanyl intravenously (IV) for pain relief, and he underwent HBOT for 90 minutes daily. Upon transfer to our hospital, he continued and completed the antibiotic course, and he received symptomatic treatment and anticoagulation therapy. In terms of the rash, he was reviewed by plastic surgery and underwent debridement of both lower limbs due to infected superficial skin necrosis. The collections from the debridement showed numerous white blood cells, without identified organisms. Intra-operative ultrasonography showed soft tissue swelling, without any subcutaneous fluid collections. The patient's clinical condition improved drastically, and the fever subsided on day 6 of admission. The patient continued undergoing HBOT to manage the skin necrosis. He underwent a total of 35 sessions of HBOT, which resulted in a signiﬁcant improvement in the rash. Ultimately, the patient was discharged home and followed up on an outpatient basis. His wounds have healed and left hyperpigmented scars with no evidence of ulceration or hypertrophy, which responded well to the keratinocyte cultured graft. He is currently using silicone gel sheets, intending to achieve mature scars within one year.

## Discussion

PF is a rare complication of *N. meningitidis* septicaemia in adults, characterized by DIC, microvascular thrombosis, and haemorrhagic skin necrosis [3]. The pathophysiology of PF involves an overwhelming inflammatory response triggered by meningococcal endotoxins, which would consequently lead to massive cytokine release, endothelial dysfunction, and subsequent coagulation cascade activation [1]. These processes result in thrombosis of dermal and systemic vasculature, progressing to purpuric skin lesions, multi-organ failure, and high mortality rates [4]. In our case, the patient presented with fever, hypotension, and rapidly evolving purpuric skin lesions, a hallmark of acute meningococcal PF. These clinical features can initially mislead medical professionals, as they can resemble viral exanthems or vasculitis. Early recognition and intervention are crucial, as PF is associated with mortality rates of up to 60% if untreated [5]. Immediate administration of broad-spectrum intravenous antibiotics, particularly third-generation cephalosporins such as ceftriaxone or cefotaxime, is the mainstay of treatment and significantly improves outcomes [6]. Additionally, hemodynamic support with intravenous fluids and vasopressors is pivotal in managing septic shock. This case serves as an illustration of fulminant meningococcal sepsis, as the patient presented with both features of shock and DIC.

These pathological mechanisms are primarily driven by the endotoxin that is produced by the meningococcal species. Concurrently, microvascular damage occurs, leading to consumption coagulopathy, which will in turn manifest as cutaneous hemorrhagic lesions. The endotoxin produced by *Neisseria* triggers a "Shwartzman-like reaction," which is an inflammatory necrotizing response that would lead to extensive vascular damage, endothelial cell apoptosis, and thrombosis [5]. The severe prothrombotic state in PF necessitates anticoagulation therapy, though its use remains controversial due to the concurrent risk of haemorrhage. Some studies suggest the benefit of protein C replacement or heparin therapy in mitigating microvascular thrombosis and reducing limb amputation rates [7]. However, in severe cases with extensive necrosis, surgical interventions, including fasciotomies or amputations, may be required to prevent further systemic complications [5].

In terms of the management of PF, there is a lack of definitive treatment guidelines. However, treatment mainly involves identifying and treating the underlying cause promptly, along with supportive management and pain control. Other therapies include HBOT, fresh frozen plasma, protein C, plasma exchange, and IVIG [2]. HBOT has been explored as an adjunctive treatment in PF due to its potential to enhance tissue oxygenation, reduce inflammation, and inhibit further microvascular thrombosis. HBOT works by increasing oxygen delivery to ischemic tissues, promoting angiogenesis, and reducing bacterial toxin activity [7]. Studies suggest that early initiation of HBOT in meningococcal PF may help mitigate necrosis and decrease the need for limb amputations by improving microcirculatory perfusion [8]. Nonetheless, more large-scale clinical trials are needed to establish definitive treatment protocols, but HBOT remains a promising therapeutic strategy in managing the severe complications of PF. Protein C, a vitamin K-dependent anticoagulation protein, has a fundamental role in regulating thrombin generation. Thus, a deficiency in protein C, whether acquired or congenital, is associated with an increased risk of PF [8]. Although pediatric findings may not be directly generalizable to adults, this is elucidated further in a retrospective study, which entailed 94 pediatric patients who had been treated with human, non-activated protein C concentrate for PF. A total of 79.8% of the patients demonstrated recovery or improvement in their PF symptoms, and there was a reduction in the need for skin grafts and amputations [8]. Although these findings are suggestive that protein C is a promising therapeutic option for PF, further randomized controlled trials in adults are needed in order to establish a definitive treatment protocol and to evaluate its long-term outcomes.

Previous studies have also highlighted the pathophysiologic mechanisms of PF and the role of surgical and adjunctive therapies [4,5]. The use of hyperbaric oxygen has shown beneficial effects in tissue recovery [6,7], while protein C concentrate remains an important therapeutic option in severe pediatric cases [8].

## Conclusions

This case highlights the successful management of a 25-year-old male patient presenting with *N. meningitidis* septicaemia and PF using HBOT as an adjunctive treatment. While early antibiotic therapy and supportive care remain the cornerstone of treatment, the use of HBOT contributed to limiting the progression of tissue necrosis, reducing the need for extensive surgical intervention, and promoting wound healing. This case supports emerging evidence that HBOT can play a valuable role in the multidisciplinary management of PF by improving tissue oxygenation, modulating the inflammatory response, and enhancing tissue salvage. Early recognition and prompt initiation of advanced therapies like HBOT may improve outcomes in severe, rapidly progressing cases. Further studies are needed to establish standardized protocols for HBOT in this setting.
